# Time series models show comparable projection performance with joinpoint regression: A comparison using historical cancer data from World Health Organization

**DOI:** 10.3389/fpubh.2022.1003162

**Published:** 2022-10-14

**Authors:** Jinhui Li, Nicholas B. Chan, Jiashu Xue, Kelvin K. F. Tsoi

**Affiliations:** ^1^JC School of Public Health and Primary Care, The Chinese University of Hong Kong, Shatin, Hong Kong SAR, China; ^2^SH Big Data Decision Analytics Research Centre, The Chinese University of Hong Kong, Shatin, Hong Kong SAR, China

**Keywords:** aging, cancer, public health, time series (TS) model, modeling

## Abstract

**Background:**

Cancer is one of the major causes of death and the projection of cancer incidences is essential for future healthcare resources planning. Joinpoint regression and average annual percentage change (AAPC) are common approaches for cancer projection, while time series models, traditional ways of trend analysis in statistics, were considered less popular. This study aims to compare these projection methods on seven types of cancers in 31 geographical jurisdictions.

**Methods:**

Using data from 66 cancer registries in the World Health Organization, projection models by joinpoint regression, AAPC, and autoregressive integrated moving average with exogenous variables (ARIMAX) were constructed based on 20 years of cancer incidences. The rest of the data upon 20-years of record were used to validate the primary outcomes, namely, 3, 5, and 10-year projections. Weighted averages of mean-square-errors and of percentage errors on predictions were used to quantify the accuracy of the projection results.

**Results:**

Among 66 jurisdictions and seven selected cancers, ARIMAX gave the best 5 and 10-year projections for most of the scenarios. When the ten-year projection was concerned, ARIMAX resulted in a mean-square-error (or percentage error) of 2.7% (or 7.2%), compared with 3.3% (or 15.2%) by joinpoint regression and 7.8% (or 15.0%) by AAPC. All the three methods were unable to give reasonable projections for prostate cancer incidence in the US.

**Conclusion:**

ARIMAX outperformed the joinpoint regression and AAPC approaches by showing promising accuracy and robustness in projecting cancer incidence rates. In the future, developments in projection models and better applications could promise to improve our ability to understand the trend of disease development, design the intervention strategies, and build proactive public health system.

**What is already known on this subject?** 

- Joinpoint regression and average annual percentage change (AAPC) are common approaches for cancer projection.
- Time series models, traditional ways of trend analysis in statistics, were less popular.
- However, there has been a lack of systematic comparison between the two common approaches and time series models in projections of cancer incidence rates.

**What this study adds** 

- A time series model, autoregressive integrated moving average with exogenous variables (ARIMAX), outperformed, in terms of accuracy and robustness, the joinpoint regression and AAPC approaches not only in long-term projections of cancer incidence rates, which was well expected, but also in short-term projections.
- Being less sensitive to the variation of incidence rates, ARIMAX produces stationary results when the incidence rates were of large variation, while joinpoint regressions and AAPC tend to produce unstable forecasts under volatile variation.
- It is noteworthy to consider using ARIMAX, or other times series models, in cancer trend projection.

## Highlights

- Cancer incidences projection using joinpoint regression and AAPC used to be common approaches rather than time series modeling
- Time series modeling projection results into comparable performance in both long-term and short-term age-related cancer incidence projection
- Normalized mean-squared-error (NMSE) over the projection period and percentage error are factor of comparison
- ARIMAX outperformed Joinpoint regression and AAPC on projecting cancer incidence rate with higher accuracy and higher volatility capacity

## Introduction

As a leading cause of death worldwide, cancer has accounted for around 10 million mortality in 2020, which equals to almost one in six deaths (1, 2). Based on the statistics of Global Cancer Observatory in World Health Organization, lung cancer, prostate cancer, and colorectum cancer are the top three cancer types with higher age-standardized incidence and mortality rates in 2020 all around the world, while the top three cancer types for women were breast, colorectum, and lung cancers (3). Apart from imposing a major threat to public health, cancer also imposes a heavy economic burden to governments and a long-lasting challenge to policymakers (4). Cancer produces the most economic loss among the world's 15 primary causes of mortality, and a recent study focusing on the global costs, health benefits, and economic benefits of scaling up treatment and imaging modalities for survival of 11 cancers indicated that $232·9 billion (95% UI 85·9–422·0) are estimated to spend between 2020 and 2030 (representing a 6·9% increase in the cost of cancer treatment) (5). Therefore, the investigation of cancer trends and the projection of cancer incidence and/or mortality have been considered as one of the most important topics in epidemiology. The construction of cancer incidence projection models is deemed necessary for policymakers to plan and prioritize their healthcare resources on cancer prevention (6–9).

A number of statistical models had been proposed for projecting cancer incidences. The joinpoint model was suggested for cancer projection by Hankey et al. (10), and employed by numerous studies (11–15). The National Cancer Institute (NCI) had promoted applications of this method by developing a free statistical software, Joinpoint, for the purpose of fitting joinpoint regression models (16, 17). Based on the software, another model, named delay-adjusted average annual percentage change (AAPC), was proposed by Clegg et al. to project cancer trends (18). The utility of this method was also demonstrated through the work of many researchers (19–21). Apart from the above-mentioned methodologies, some researchers applied autoregressive integrated moving average (ARIMA) models, or their variants such as ARIMA with exogenous variables,to project cancer trends (22–25). Popularized by Box and Jenkins in the 1970s (26). the ARIMA model is considered to be one of the classical approaches to analyze time series data in statistics. It has demonstrated success in tackling various time trend prediction problems such as economic cycles (27), foreign exchange markets (28), traffic volumes (29), and visits to emergency department (30). However, its capability for cancer projection has seldom been explored in comparison to other projection methodologies. Furthermore, ARIMA model was sometimes criticized for being an outdated model and some suggested that there exist other models that could outperform ARIMA (31, 32).

Most of the previous research papers applied various cancer projection methods, but there was no consensus among researchers regarding the best methodology. This paper aims to answer the question by comparing short-term and long-term projection performances of three projection methodologies, including joinpoint regression, AAPC, and Autoregressive Integrated Moving Average with exogenous variable (ARIMAX).

## Methods

### Data source

Cancer data from worldwide cancer registries were extracted from the dataset Cancer Incidence in Five Continents time trends, CI5plus, from the International Agency for Research on Cancer under the World Health Organization (33). Promoting international collaboration in cancer-related research, the Agency made the cancer data available for free download in the World Wide Web (http://ci5.iarc.fr/CI5plus). The annual cancer incidence of 28 types in 118 regions/countries along with their respective population sizes until 2007 were included. The population and incidence data were classified by sex and presented in five-year age groups.

### Data preparation

Although data of a total of 28 types of cancers were available in the cancer registries, only aging-related cancers were included in this study, including bladder, colorectal, esophagus, lung, pancreas, prostate, and stomach cancers (22). This was because the incidence rates of various cancers in young age groups were generally lower than those in the elder groups. Sixty-six regions from 31 countries with at least 25 years of cancer data were included in this study. The total population size of these regions is over 240 million in 2007^1^, in which 31 million (13.0%) are aged 65 or above. A list of cancer registries available in the dataset along with their respective population aged 65 years or above is shown in Table 1.

**Table 1:** List of cancer registries from the World Health Organization^a^.

**Registries with** ** < 25 years of cancer data (*****n*** **=** **36)**	
Austria, Tyrol (107,907)	Austria, Vorarlberg (52,539)
Brazil, Goiania (69,209)	China, Jiashan County (49,126)
China, Shanghai (943,575)	Croatia (762,633)
Ecuador, Quito (83,632)	France, Haut-Rhin (113,357)
France, Herault (175,143)	India, Poona (201,894)
Italy, Ferrara Province (90,835)	Italy, Florence (273,949)
Italy, Modena (141,551)	Italy, Romagna (266,404)
Italy, Sassari Province (85,253)	Italy, Torino (215,253)
Japan, Nagasaki Prefecture (361,143)	Latvia (375,861)
Malta (56,519)	Russia, St Petersburg (741,504)
Spain, Albacete (68,533)	Spain, Cuenca (48,980)
Spain, Girona (109,316)	Spain, Granada (141,050)
Switzerland, Graubunden and Glarus (38,233)	Switzerland, Valais (47,729)
Switzerland, Vaud (102,353)	Thailand, Lampang (84,248)
Thailand, Songkhla (100,106)	The Netherlands (2,392,589)
Uganda, Kyadondo County (31,043)	UK, East of England Region (950,840)
UK, England, Oxford (399,970)	UK, Northern Ireland (243,305)
USA, New Jersey (1,133,775)	USA, New York State (2,554,067)
**Registries with 25 to 29 years of cancer data (*****n*** **=** **25)**	
Australia, New South Wales (941,945)	Australia, Queensland (511,192)
Australia, Victoria (702,719)	Australia, Western Australia (250,822)
China, Hong Kong (872,200)	Colombia, Cali (156,603)
Costa Rica (267,939)	Czech Republic (1,495,670)
France, Isere (170,688)	France, Somme (87,946)
France, Tarn (80,695)	India, Chennai (Madras) (278,295)
Italy, Ragusa Province (56,382)	New Zealand (526,370)
Philippines, Manila (193,590)	Poland, Cracow City (112,974)
Spain, Murcia (192,733)	Spain, Tarragona (118,522)
Switzerland, Neuchatel (29,911)	Switzerland, St Gall-Appenzell (83,418)
Thailand, Chiang Mai (140,364)	UK, England, Birmingham and West Midlands Region (884,910)
UK, England, North Western (1,059,140)	UK, England, South and Western Regions (1,283,450)
UK, England, Yorkshire (619,210)	
**Registries with 30 to 39 years of cancer data (*****n*** **=** **28)**	
Australia, South Australia (241,067)	Australia, Tasmania (73,021)
Canada, Nova Scotia (140,527)	France, Bas-Rhin (156,314)
France, Calvados (107,465)	France, Doubs (80,033)
Germany, Saarland (225,429)	India, Mumbay (Bombay) (644,857)
Italy, Lombardy, Varese province (171,194)	Italy, Parma (98,012)
Japan, Miyagi Prefecture (493,652)	Lithuania (530,213)
Spain, Navarra (105,720)	Switzerland, Geneva (66,511)
The Netherlands, Eindhoven (153,172)	UK, England, Merseyside and Cheshire (397,670)
UK, Scotland (845,618)	USA, California, Los Angeles (961,743)
USA, California, San Francisco (393,541)	USA, Connecticut (463,141)
USA, Georgia, Atlanta (252,492)	USA, Hawaii (179,840)
USA, Iowa (437,787)	USA, Michigan, Detroit (482,123)
USA, New Mexico (247,996)	USA, SEER (nine registries) (3,345,868)
USA, Utah (234,836)	USA, Washington, Seattle (502,414)
**Registries with 40 to 49 years of cancer data (*****n*** **=** **7)**	
Canada, Saskatchewan (149,988)	Estonia (229,813)
Israel (593,700)	Japan, Osaka Prefecture (1,422,146)
Singapore (305,600)	Slovakia (644,927)
Slovenia (292,034)	
**Registries with at least 50 years of cancer data (*****n*** **=** **6)**	
Canada, Manitoba (154,392)	Denmark (804,353)
Finland (695,082)	Iceland (31,184)
Norway (696,600)	Sweden (1,542,834)

^**a**^Numbers in parentheses represent the population of aged 65 years or above in 2007. The latest available population was shown instead if the population in 2007 was unavailable. Registries with < 25 years of cancer data were excluded in the study.

### Projection methods

In this study, three projection models fitted using data of the seven aging-related cancers were included for each region. The models included (i) Joinpoint regression; (ii) AAPC; and (iii) ARIMAX. Applying weighted least square estimate with linear regression through existing data points, joinpoint regression is frequently utilized for retrospective studies and risk factor analyses, but its adjustment and regression capabilities are limited for forecasting. Consequently, investors sometimes apply AAPC as a geometrically weighted average of the multiple annual percentage changes (APCs) from the joinpoint regression analysis, with time-segment weight across the selected time period (18, 34). It offers superior predictive ability compared to joinpoint regression. Nonetheless, the AAPC foresting variation could expand as the foresting period continues. We would like to determine if ARIMAX could circumvent the limitations of joinpoint and AAPC for public health trend forecasting in the situation of few data records or delayed records, which frequently occur in cancer forecasting. ARIMAX is an extension of the univariate autoregressive model used to forecast a vector of time series. In addition to standard time dependence, it employs vector auto-regression, and the dynamic observation improved projection accuracy.

**Figure 1:**
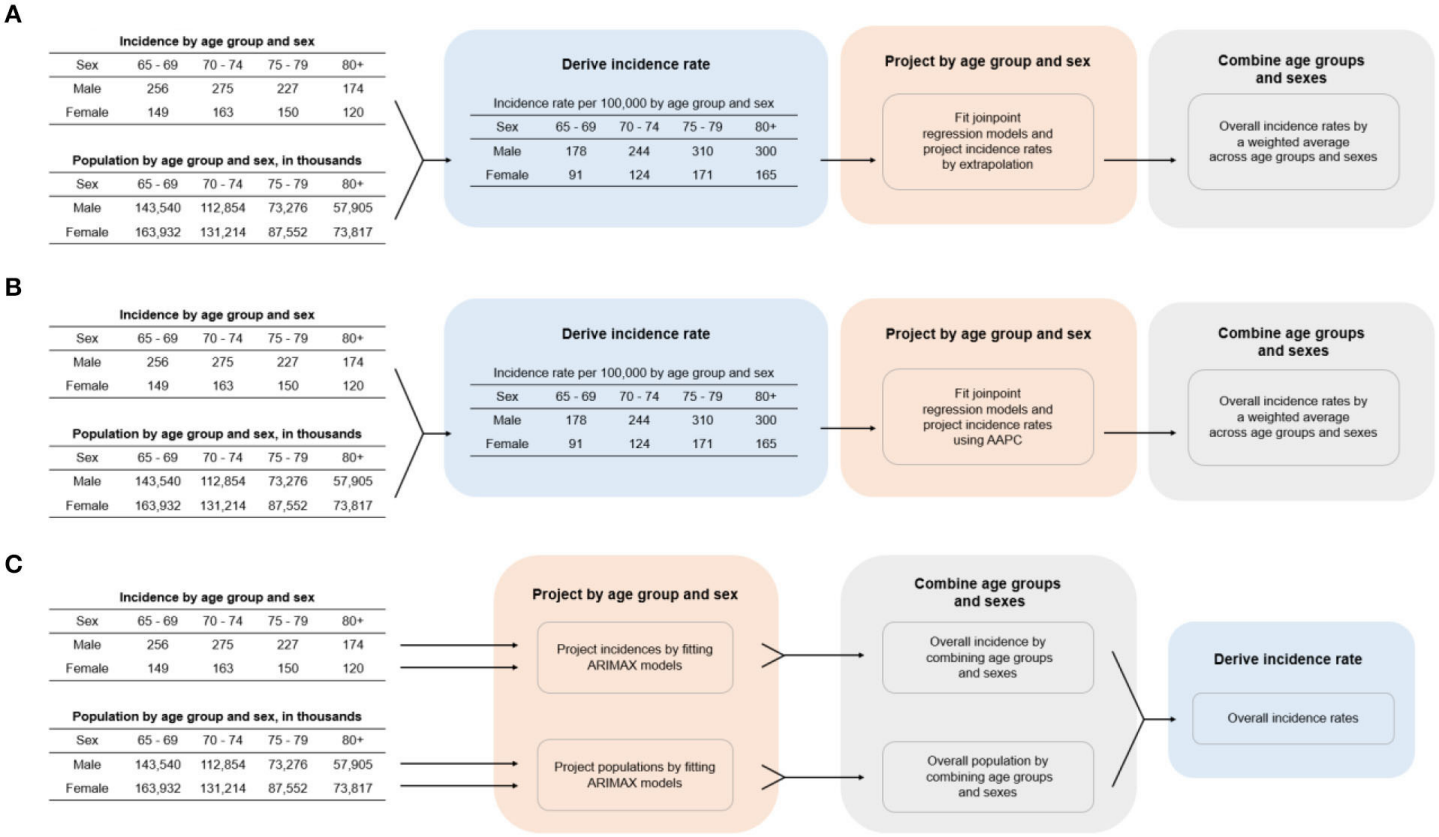
Procedures for calculating cancer incidence rate for the three projection methods. **(A)** Joinpoint regression, **(B)** AAPC, **(C)** ARIMAX. AAPC, average annual percentage change; ARIMAX, autoregressive integrated moving average with exogenous variables.

The population was stratified into different sexes and age groups and their incidence rates were projected separately. The overall incidence rates were combined from both male and female incidence rates, weighted by their respective sample sizes. The projection models were derived from incidence and population figures of the first 20 available years, while data from the subsequent years served for the purpose of validation. Model validation time period intervals varied across different regions due to different available years of data records in each region, but the time periods of all validation data used were at least five years. The three methods are described below with more detail, while a brief illustration of the procedures in all three approaches is also available in Figure 1.

### Joinpoint regression

Joinpoint regression models consist of linear segments of different slopes connected at joinpoints, allowing flexibility in the rate of change of the response in time (17). Joinpoint regression models with the log-transformed cancer incidence rate as response were fitted in this approach. In particular, the number of joinpoints (or changepoints or breakpoints) in each regression model was automatically decided by NCI's trend analysis software Joinpoint through using a grid search algorithm (16, 35). Given that sample size of our training data was limited to 20, the maximum number of possible joinpoints was constrained to two. In other words, the fitted models could be illustrated by plotting log-transformed incidence rate against time. These graphs consisted of one to three continuous straight-line segments that were connected together at the joinpoints. Visually speaking, projecting future incidence rates were the same as extending the rightmost line segment to the desired time points.

### AAPC

NCI suggested that the delay-adjusted AAPC could be useful when summarizing a trend in incidence rate over a fixed period, where the segmentation of time was determined by fitting an underlying joinpoint regression model (16). Incidence rates were projected based on the AAPC by a method proposed by Rahib et al. (20). For each cancer, region, and sex, a baseline incidence rate and its respective AAPC were determined after fitting the same joinpoint regression model described above. The baseline incidence rate ***I***_*****d*****_ was determined by averaging the incidence rates of the last 3 years of the 20-year model training period. Adjusting ***I***_*****d*****_ by AAPC gave the final projected incidence. This could be described as


$$\begin{array}{l} Projected\;Incidence=\{I_{d}\times {\left(\frac{AAPC}{100}+1\right)}^{n}\;,\;for\;AAPC>0;\\ \;I_{d}\times {\left(\frac{\left|AAPC\right|}{100}+1\right)}^{-n},\;for\;AAPC<0\} \end{array}$$

where ***n*** is number of years elapsed since the end of the model training period.

### ARIMAX

This method was replicated from a previous study that investigated the relationship between incidence of colorectal cancer and aging populations (22). Two individual ARIMAX models were built to project incidence and population, respectively. The number of years from the starting year of the available data for the concerned region, represented by ***t***, was used as an exogenous variable in the model. Mathematically, the models could be represented as


$$\begin{array}{l} X_{t}=\phi X_{t-1}+\theta \epsilon_{t-1}+\epsilon_{t}+\alpha +\beta \;t \end{array}$$

where ***X***_*****t*****_ can be either the incidence or the population and ϵ_*****t*****_ is the white noise, both at time ***t***, and ***X***_*****t***−1**_ and ϵ_*****t***−1**_ are their respective lagged variables at time ***t***-1. During ARIMAX modeling, the dominant predictor variable is the time factor. We organized our dataset and noted incidence case in each year time interval, and then fit the observed data in the ARIMAX fit and choose the projection lagged period from training data. The parameters of each ARIMAX model were estimated by the corresponding data in the training period. In case the time series did not exhibit any identifiable ARIMA structure, the model would reduce to a simple linear regression model. The predicted incidence rate at time ***t*** was obtained by dividing the projected incidence by the projected population. The model was conducted by R statistical software (version 4.0.3).

### Model comparison

Three, 5, and 10-year projection periods were considered in this study to investigate the strengths and weaknesses of the models in projecting short-term and long-term cancer trends. Afterwards, two model accuracy metrics were computed for each period to evaluate the performances of the three methods. They were normalized mean-squared-error (NMSE) over the projection period and percentage error at the end of the projection period. However, the 10-year accuracy metrics for some registries cannot be computed due to insufficient real data from the projection period. Thus, only three-year and five-year metrics were computed for those registries. NMSE quantified the overall magnitude of the projection error in the given period while percentage errors enabled us to evaluate both the relative magnitude and the direction of projection error at the given time point. With the two defined accuracy metrics, the best method was identified by (i) a percentage error that was closest to zero; or (ii) the smallest NMSE. Two aggregations were made to evaluate the overall performances of the three methods. First, the accuracy metrics were aggregated across all registries using the population aged 65 years or above of the last available year as weights. Conventionally, it has been defined the old age as the duration of life beginning at the age of 65 years old, with established age ranges of young old (65–74 years), middle old (75–84 years), and extremely elderly (≥85 years) (36, 37). Studies have approved that that aging-related cancers are cancers with incidence rates dominated by the population aged 65 or above, and with no signs of their incidences shifting to the younger generation (22, 38). This enabled us to evaluate the overall performance of the different methods by different cancer types, gender, and countries. Second, the accuracy metrics were averaged across the seven chosen cancers, and then weighted averages were taken across registries from the same country. This produced by-country accuracy metrics for our comparison. To compare the methods across all countries and cancers as a whole, a weighted average error for each metric was also computed using the population of each group as weights. The weighted average of the between the positive and negative percentage was calculated by averaging the percentage error projections for each cancer and multiplying by the weight of each cancer cohort size.

## Results

**Figure 2:**
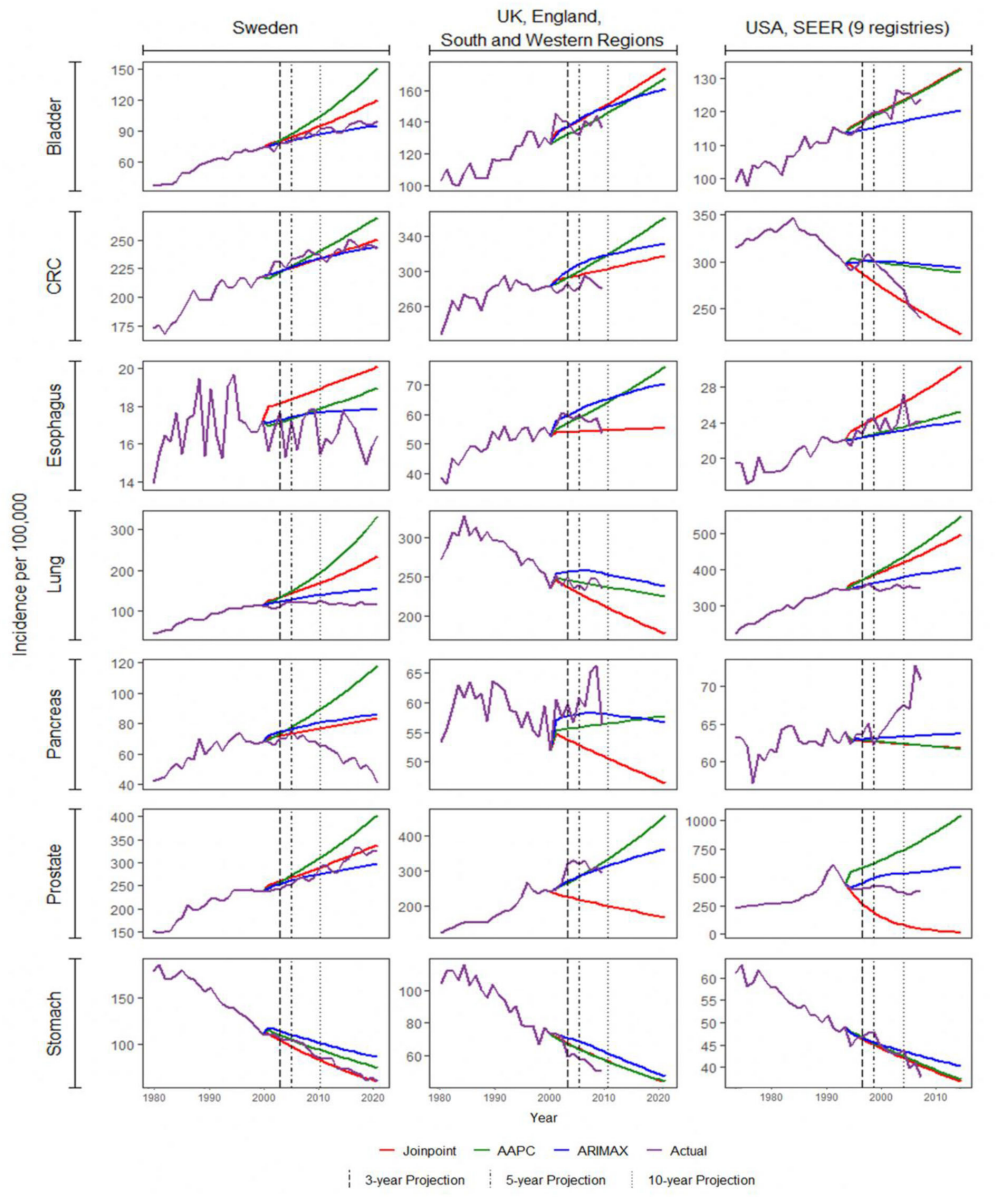
Projection of cancer incidence for Sweden, UK, and US. AAPC, average annual percentage change; ARIMAX, autoregressive integrated moving average with exogenous variables; CRC, colorectal cancer.

All three methods were used to model the incidence rates of the seven types of cancer from 66 regions. This resulted in a total of 462 cases/combinations. Three, five, and 10-year projections were obtained from the fitted models. The corresponding accuracy metrics for each method were computed in all cases, except for the 10-year projection of 25 regions due to unavailability of actual data. To illustrate any difference in performances of the methods, the actual and projected incidence rates of the seven cancers in Sweden, the United Kingdom, and the United States^2^ were plotted in Figure 2. These registries were chosen as they were three of the largest registries. Among most of the 27 cases there, projections of the three methods were close to one another, and the changes in projected incidence shared the same direction. The projections deviated for some cancers such as lung and prostate cancers. For prostate cancer, the difference was indeed substantial. The changes in projected incidence rates in the UK and the US by the three methods were not even in the same direction, with the intermediate result among the three obtained by ARIMAX.

Among all combinations of cancers and registries, ARIMAX performed the best in 185 (40.0%) cases in terms of five-year percentage error, 179 (38.8%) in terms of five-year NMSE, 131 (45.6%) in terms of 10-year percentage error, and 121 (42.2%) cases in terms of 10-year NMSE. On the other hand, joinpoint regression and AAPC, respectively, performed the best in 125 (27.1%) and 152 (32.9%) cases in terms of 5-year percentage error, 127 (27.5%) and 156 (33.8%) in terms of 5-year NMSE, 84 (29.3%) and 72 (25.1%) in terms of 10-year percentage error, and 81 (28.2%) and 85 (29.6%) in terms of ten-year NMSE. According to these four-accuracy metrics, the ARIMAX stood out from the three approaches on more occasions.

The performance characteristics of the three methods by cancer are illustrated in Table 2. Small absolute percentages correspond to good projections. As far as three-year projection was concerned, the ARIMAX was on a par with AAPC in giving the least NMSE in three out of seven cancers. ARIMAX gave the least percentage error in four out of seven cancers and outperformed the other two methods. When 5-year projection was considered, AAPC was the best in giving the least NMSE in four out of seven cancers, while ARIMAX gave the least percentage error in four out of seven cancers. In regard to ten-year projection, ARIMAX was on a par with AAPC in giving the least NMSE in three out of seven cancers. Once again, ARIMAX stood out, attaining the least percentage error in five out of seven cancers. With reference to a single weighted average across all seven cancers, ARIMAX yielded the least NMSEs or percentage errors in five out of six scenarios. Based on the current results, ARIMAX was relatively superior to the other two methods, while joinpoint regression was kind of worst. Additionally, the validation results of each model by different gender groups were displayed in Supplementary Table 3, which was consistent with the main findings in Table 2.

**Table 2:** Comparison between actual and projected cancer incidence.

**Cancer**	**Normalized mean-squared-error**			**Percentage error** ^**a**^		
**(Total incidence^b^)**	**over the period** ^**a**^					
	**Joinpoint**	**AAPC**	**ARIMAX**	**Joinpoint**	**AAPC**	**ARIMAX**
**In 3-year projection**						
Bladder (31,648)	3.9%	2.8%	**2.3%**	6.3%	4.9%	**1.1%**
CRC (85,600)	0.9%	**0.7%**	1.1%	3.9%	3.5%	**1.6%**
Esophagus (9,597)	4.1%	**2.2%**	2.5%	3.7%	3.0%	**1.8%**
Lung (83,767)	0.9%	0.7%	**0.6%**	4.7%	4.6%	**1.2%**
Pancreas (19,823)	1.8%	**1.1%**	1.2%	2.0%	**0.3%**	−2.6%
Prostate (68,536)	4.8%	2.1%	**2.1%**	**−2.2%**	−3.5%	−8.3%
Stomach (22,613)	**1.1%**	1.3%	1.3%	**0.7%**	4.2%	4.2%
**Weighted average**	2.4%	**1.5%**	1.6%	3.8%	3.7%	**3.4%**
**In 5–year projection**						
Bladder (31,648)	6.0%	4.4%	**3.7%**	14.6%	13.3%	**7.9**%
CRC (85,600)	1.1%	**0.9%**	1.4%	3.5%	3.8%	**0.8%**
Esophagus (9,597)	5.8%	**2.3%**	3.0%	2.1%	1.2%	**0.3%**
Lung (83,767)	1.4%	1.0%	**0.8%**	7.3%	8.5%	**3.0%**
Pancreas (19,823)	2.4%	**1.5%**	1.6%	0.8%	**−0.6%**	−4.8%
Prostate (68,536)	7.8%	**3.0%**	3.0%	1.0%	**−0.5%**	8.6%
Stomach (22,613)	**1.3%**	1.6%	1.8%	**0.8**%	4.9%	6.5%
**Weighted average**	3.5%	2.1%	**2.1%**	6.5%	6.6%	**5.3%**
**In 10-year projection**						
Bladder (21,411)	9.8%	5.0%	**2.9%**	21.8%	18.9%	**3.1%**
CRC (53,414)	2.0%	**1.3%**	1.8%	8.2%	9.9%	**1.5%**
Esophagus (5,541)	14.5%	**3.1%**	3.4%	6.1%	2.7%	**0.2%**
Lung (55,601)	3.6%	4.5%	**1.6%**	21.7%	29.7%	**8.1%**
Pancreas (13,344)	4.3%	2.8%	**1.9%**	12.1%	12.4%	**0.7%**
Prostate^c^ (35,827)	25.7%	**5.5%**	6.4%	21.6%	**1.7%**	−18.0%
Stomach (15,713)	**1.3%**	1.7%	2.1%	**0.0%**	4.5%	7.8%
**Weighted average**	7.8%	3.3%	**2.7%**	15.0%	15.2%	**7.2%**

AAPC, average annual percentage change; ARIMAX, autoregressive integrated moving average with exogenous variables.

^a^The projection errors were compared across the three methods, and the one with the least error was bolded.

^b^As of the latest available year per registry. Sixty-six registries were included for 3-year and 5-year projections while 41 registries were included for 10-year projections, resulting in different fewer total incidences for 10-year projection.

^c^The incidences of prostate cancer from the United States were excluded in 10-year projection as all three methods produced poor results and resulted in exceptionally large error rates.

In line with the previous research on the relationship between the incidence of colorectal cancer and aging populations (22), Table 3 presented a comparison of the three approaches based on ten-year projections of colorectal cancer incidence over 23 relatively well-developed countries from the 31 countries in Table 1. Analogous comparisons concerning stomach cancer and prostate cancer were presented in Supplementary Tables 1, 2 respectively. As in Table 2, NMSE and percentage errors in projections were displayed as the criteria. The smaller an absolute percentage, the better the projection. ARIMAX stood out marginally from the three approaches in ten-year projection with respect to either NMSE or percentage error. It yielded the least absolute percentages in nine out of 23 projections for the chosen countries. In comparing the three methods by a single weighted average of the 23 NMSEs in projection, AAPC yielded the least weighted average percentage (1.3%), while the averages (2.0% for joinpoint regression and 1.8% for ARIMAX) by the other two methods were comparable and relatively close. Similar results applied in terms of the weighted average of percentage errors across the 23 countries. The average error by AAPC is 14.4% compared with 17.2% by joinpoint regression and 15.5% by ARIMAX.

**Table 3:** Comparison between actual and 10-year projected incidence on colorectal cancer.

**Country**	**Normalized mean-squared-error**			**Percentage error**		
**(Total incidence^b^)**	**over the Period** ^**a**^			**at the tenth year** ^**a**^		
	**Joinpoint**	**AAPC**	**ARIMAX**	**Joinpoint**	**AAPC**	**ARIMAX**
Australia (1,215)	1.0%	1.0%	**0.6%**	**4.4%**	5.3%	−6.3%
Canada (1,544)	**0.8%**	1.1%	0.9%	11.0%	10.8%	**3.3%**
Denmark (2,851)	**0.1%**	0.1%	0.2%	2.0%	**−1.4%**	−5.3%
Estonia (528)	0.4%	**0.4%**	0.9%	**3.7%**	5.5%	−6.1%
Finland (1,752)	0.6%	**0.2%**	1.0%	8.5%	**4.2%**	−13.6%
France (904)	**3.9%**	6.1%	6.7%	**16.5%**	27.9%	20.0%
Germany (745)	2.9%	1.0%	**0.2%**	25.5%	16.7%	**1.1%**
Iceland (81)	**1.1%**	1.6%	5.2%	5.5%	**0.9%**	−19.1%
India (260)	16.8%	14.5%	**10.2%**	28.4%	25.7%	**13.6%**
Israel (2,192)	8.7%	5.6%	**0.3%**	52.2%	44.7%	**5.9%**
Italy (872)	1.9%	1.2%	**1.2%**	−11.7%	−14.3%	**−6.3%**
Japan (5,602)	3.7%	**0.9%**	4.7%	30.2%	**10.6%**	−26.1%
Lithuania (1,047)	**0.3%**	0.6%	0.9%	3.6%	**0.8%**	−1.4%
Norway (2,535)	**0.7%**	0.9%	3.0%	**−12.3%**	−13.4%	−24.8%
Singapore (878)	4.6%	1.8%	**1.2%**	42.3%	29.3%	**−5.7%**
Slovakia (2,057)	3.5%	**0.4%**	2.5%	25.0%	**3.7%**	−17.1%
Slovenia (971)	0.3%	**0.3%**	2.6%	**0.3%**	−5.1%	−23.9%
Spain (314)	1.9%	1.3%	**0.6%**	24.7%	21.8%	**−3.3%**
Sweden (4,295)	0.1%	**0.1%**	0.1%	−1.5%	**1.3%**	−1.4%
Switzerland (182)	2.7%	1.8%	**0.8%**	21.8%	17.8%	**7.6%**
The Netherlands (547)	0.6%	0.4%	**0.3%**	8.4%	5.7%	**−4.1%**
UK (3,822)	**0.3%**	0.3%	0.5%	6.9%	**6.7%**	10.0%
USA (17 947)	0.8%	**0.6%**	1.1%	**−1.7%**	12.8%	15.8%
**Weighted Average**	2.0%	**1.3%**	1.8%	17.2%	**14.4%**	15.5%

AAPC, average annual percentage change; ARIMAX, autoregressive integrated moving average with exogenous variables.

^*a*^The projection errors were compared across the three methods, and the one with the least error was bolded.

^*b*^As of the latest available year per registry.

## Discussion

In this study, ARIMAX demonstrated its promising performance in projecting cancer incidence rates. It outperformed the joinpoint and AAPC approaches for the majority of times to come extent, especially in 10-year projection. However, cautions are still required for the method adoption and results interpretation as the cancer incidence situation and changeable policy in different counties. A study reported that joinpoint and AAPC approaches should not be used for long-term projections (39), so they were not anticipated to perform particularly well. Surprisingly, they were outperformed by ARIMAX even in five-year projection. This suggested a potential weakness for the two methods. Previous studies have indicated that the results from joinpoint regressions were rather unstable if the incidence rates themselves were of large variation (11, 40), which could be due to a small population size or insufficient training data points. As AAPC is built upon joinpoint regression, it suffers from the same problem. In projections, joinpoint regression relies on magnitude and direction of the latest trend, and extrapolates the pattern indefinitely. Unless the latest trend continues or there is no drastic change in either magnitude or direction of the trend, the approach would fail in long-term projection, or even in short-term projection. In contrast, ARIMAX might be less sensitive to the variation of incidence rates as its parameters were computed by all available data points rather than only the latter ones, which ultimately leads to more accurate projections. It shall be noted that ARIMAX requires more information as inputs when compared to the others. Both joinpoint and AAPC approaches project future trends by directly using historical cancer incidence rates. ARIMAX, on the contrary, takes historical population and incidence as two separate inputs and creates two corresponding ARIMA models. Final projections were then computed by combining the two. As additional information is utilized by ARIMAX, the projections are generated with more robustness. Furthermore, we may determine, based on a survey of existing studies utilizing these projection models and approaches, that joinpoint regressions were adopted for the cervical, lung, and breast cancer mortality forecast (41–43). Another study adopted joinpoint model to calculate the AAPC of colorectal cancer mortality and incidence in Guangzhou urban residents from 1972 to 2015, illustrated good prediction of incidence and mortality rate in 2016 to 2025 by ARIMA model (44). Besides, joinpoint regression analysis and AAPC were also used to calculate the temporal trend and ARIMA model was applied to the 10-year projection of aortic aneurysm (45). Seasonal ARIMA (SARIMA) was also approved to show a strong ability in estimating the long-term epidemic treads of hemorrhagic fever with renal syndrome (HFRS) (46).

Only aging-related cancers were included in this study. As a completed unit of time, age is always used in virtually all studies of cancer epidemiology. Studies also consider cancer as an age-related disease because the increased risk with age and complex biological processes associated with aging (47). Moreover, a recent study has suggested that aging-related cancers are cancers with incidence rates dominated by the population aged 65 or above, and with no signs of their incidences shifting to the younger generation (22). Based on the statistics from John Hopkins Medicine, the incidence of developing cancer was increased 11-fold among people over 65 years old compared to the younger individuals. Moreover, cancer incidence has climbed 26% in the over-65 population during the last 30 years, compared to a 10% increase in the younger group under 65 (48). As a result, these cancers have a much larger case loading for the elder generation when compared to the younger generation. The choice of focusing on aging-related cancers is justified by the fact that incidence rates calculated by using a larger case loading tend to be more stable and more accurate. As the aim of this study is to compare three methods for cancer incidence projection, it is important to ensure our findings are based on stable results.

Projections of prostate cancer incidence in the US were excluded throughout our comparisons due to their extremely poor performances (see Supplementary Table 2). The three methods seemed to produce contrasting results in projection, as shown in Figure 2. The mean squared error and percentage error were chosen as the accuracy indicator. Such an issue was exemplified in considering different regions in the US, the UK, and India (see Supplementary Figure 1). The joinpoint regression approach tended to produce very poor projections. When the last trend was estimated to be relatively steep, its projection would become fairly extreme. This was probably due to the same deficiency we mentioned above regarding the projection of joinpoint models. Given that the incidence of prostate cancer was not far off from those of colorectal cancer and lung cancer (68,536 vs. 85,600 and 83,767 in 2007 from Table 2), it prompted some more efforts in trend analysis and projection of prostate cancer incidence.

The major limitation of this study was data coverage. To ensure the training period was at least 20 years, our study only included the countries or regions with data available for no < 25 years. As a result, most of them were western or developed countries and few belonged to developing countries. As the cancer incidence rates of developing countries were expected to be higher and more volatile, their moving trends might behave differently from those of developed countries. While our results demonstrated that ARIMAX was in general more accurate than the other two approaches, this conclusion may not be applicable to some underdeveloped nations due to health development inequalities and disparities, as well as skewed investments in public health and education. In addition, different factors including prevention strategies, lifestyle habits, and diagnostic capability etc. from different countries might affect the 10-year projection. A previous study conducted in the UGG-SMART (Utrecht Cardiovascular Cohort – Second Manifestations of ARTerial disease) cohort demonstrated that the lifetime and 10-year risk of total, colorectal, and lung cancer could be predicted with reasonable accuracy using validated prediction models with predictors such as age, sex, smoking status, weight, and other disease status, etc. (49). Another study utilizing a new prediction model to estimate the 10-year breast cancer survival rate in New Zealand demonstrated the model's high clinical utility, although the authors noted that other predictor factors during the period should also be considered in future research (50). Therefore, the interpretation of the findings should take the development of the health promotion strategies into consideration. From the independent report commissioned by World Health Organization (WHO), health aging and good health service were focused for the global health journey of 2007–2017 (47). Besides, as this study did not consider other methods such as Nordpred and Age-Period-Cohort model, it still requires more study and evidence to identify the different projection methods for their specific best epidemiological application. Currently, many short-term cancer trend projections employed joinpoint regression and AAPC, partly due to them being used and encouraged by authorities in the field. The findings here may provide insight as to which projection methods could be applied to achieve better short-term projection of cancer trends.

## Data availability statement

The original contributions presented in the study are included in the article/Supplementary material, further inquiries can be directed to the corresponding author/s.

## Author contributions

JL: conceptualization, methodology, writing-original draft, and revision. NC and JX: software, formal analysis, and validation. KT: study design, guarantors, and writing review. All authors contributed to the article and approved the submitted version.

## Conflict of interest

The authors declare that the research was conducted in the absence of any commercial or financial relationships that could be construed as a potential conflict of interest.

## Publisher's note

All claims expressed in this article are solely those of the authors and do not necessarily represent those of their affiliated organizations, or those of the publisher, the editors and the reviewers. Any product that may be evaluated in this article, or claim that may be made by its manufacturer, is not guaranteed or endorsed by the publisher.

## Footnotes

^1^Due to the basic requirement in regard to amounts of data of the projection methods under comparison, it was not possible to include more than 31 countries in this study.

^2^The three well-developed Western countries were selected as there were available data of more years (e.g., 50 years for Sweden) as well as there were relatively more population of aged 65 years or above in 2007 (e.g., more than 10, and 6 million in the US, and the UK, respectively).
