# Supplementary material for: Time series models show comparable projection performance with joinpoint regression: A comparison using historical cancer data from World Health Organization
Source: Front Public Health. 2022 Oct 14;10:1003162. doi: 10.3389/fpubh.2022.1003162 (PMC9614249; doi:10.3389/fpubh.2022.1003162)
Supplement: Supplementary file 1 [file Table_1.DOCX]

**eFigure 1. Actual and Projected Incidence of Prostate Cancer in Registries from US, UK and India**

**US** ^a^


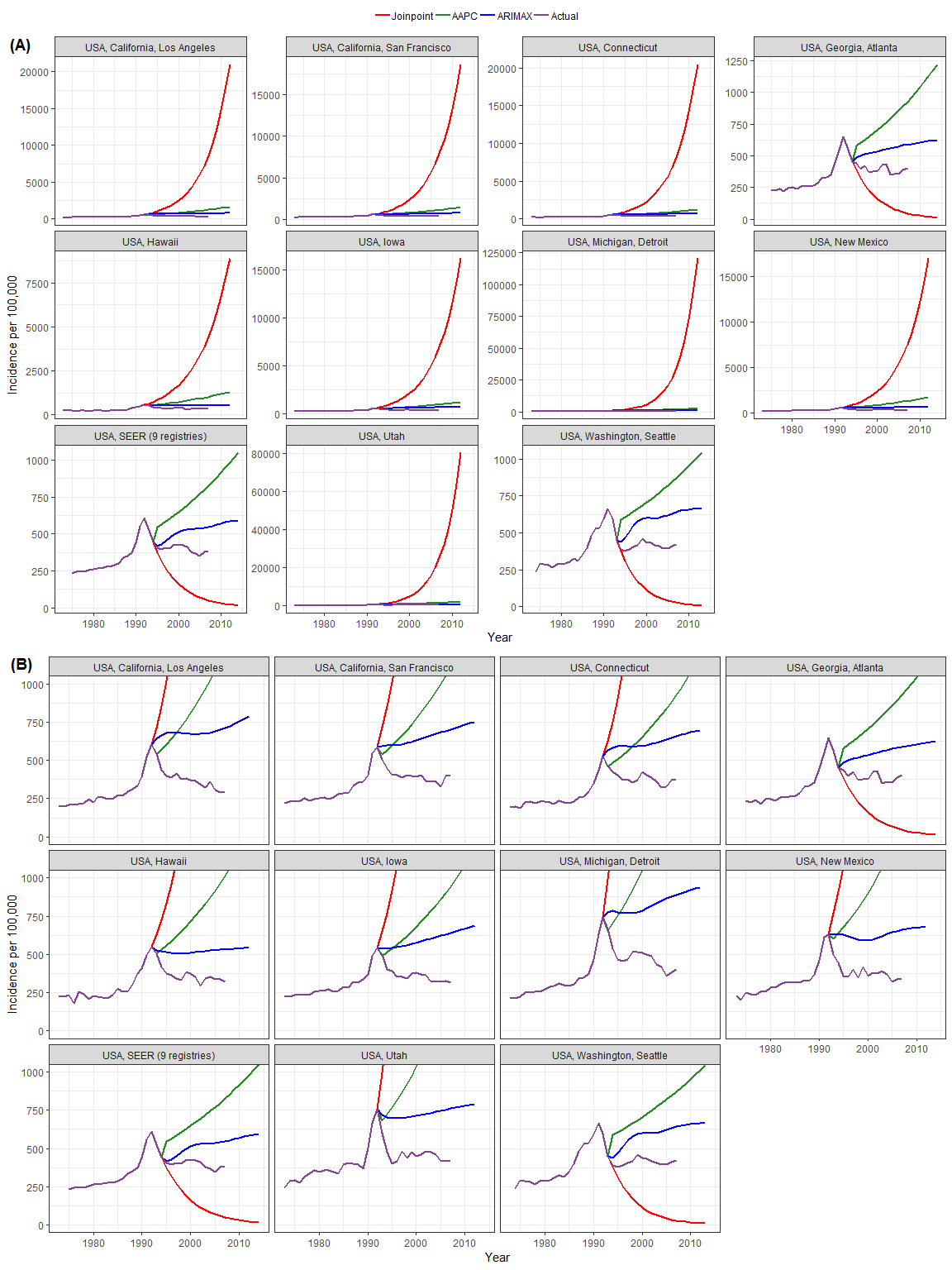


**UK** ^a^


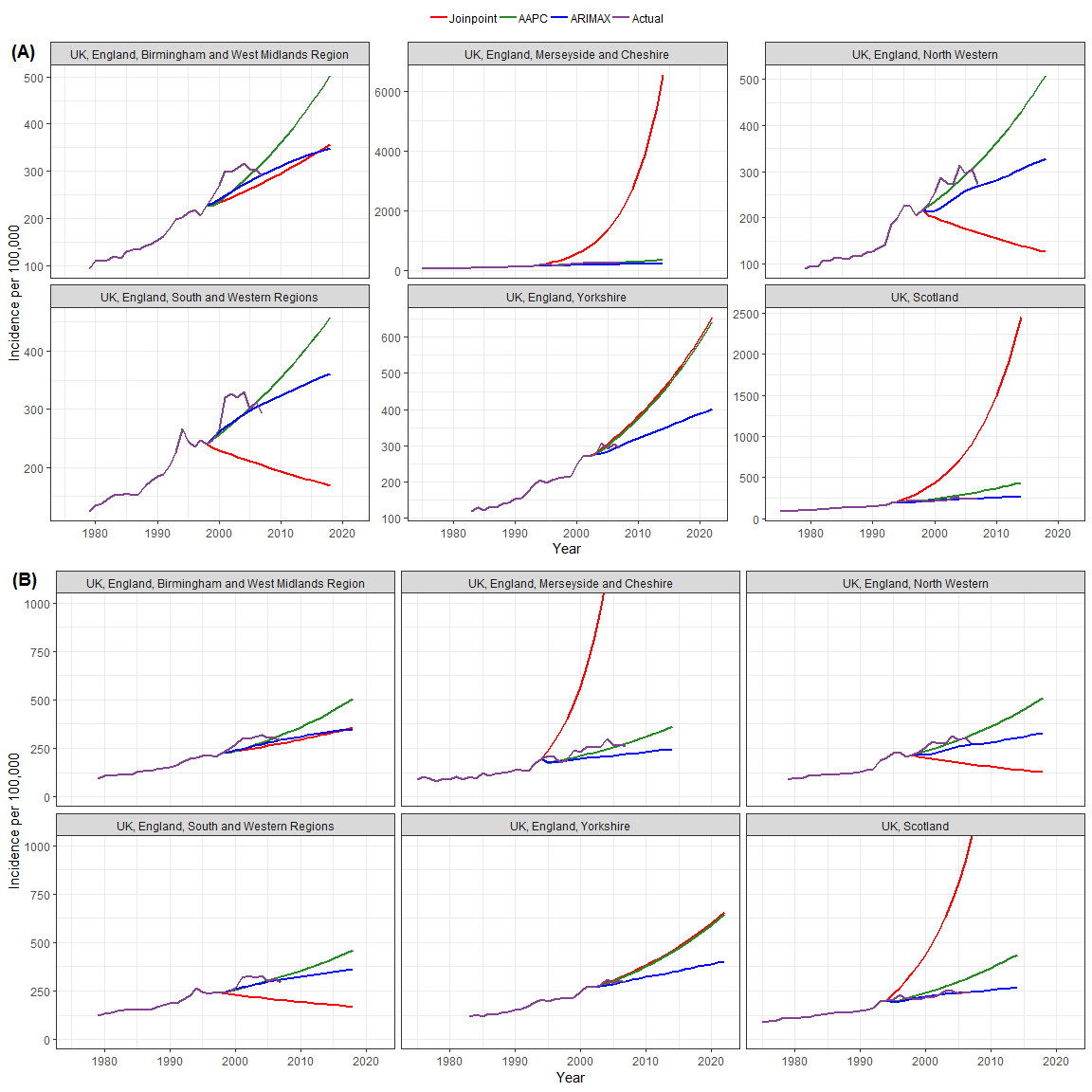


**India** ^a^


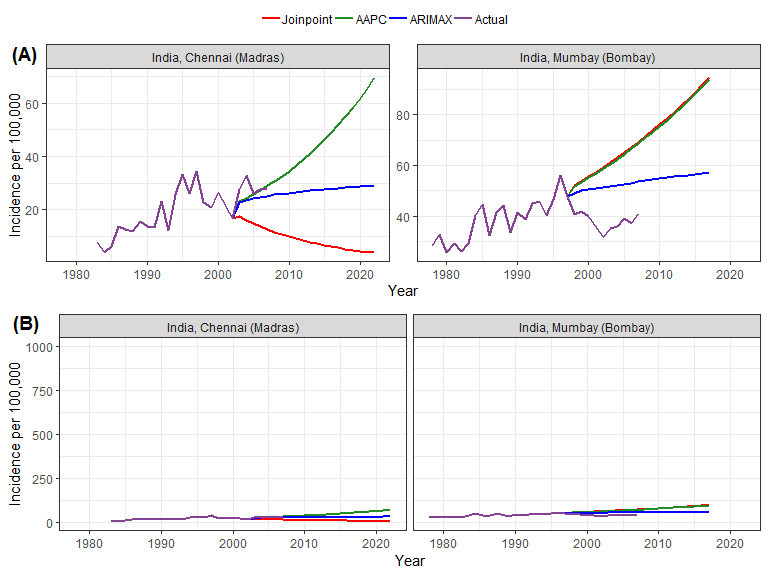


**Notes:** **Abbreviations:** AAPC, average annual percentage change; ARIMAX, autoregressive integrated moving average with exogenous variables.

^a^ (A) and (B) both showed the incidence rates of prostate cancer in a registry against time. The vertical axes of the plots in (B) were fixed to a range of incidence per 100,000 between 0 and 1,000 for the sake of comparison.

**eTable 1. Comparison between Actual and 10-year Projected Incidence on Stomach Cancer**

| **Country**  **(Total incidence**^b^**)** | **Normalized Mean-squared-error**  **over the Period**^a^ | | | **Percentage Error**  **at the Tenth Year**^a^ | | |
| --- | --- | --- | --- | --- | --- | --- |
|  | **Joinpoint** | **AAPC** | **ARIMAX** | **Joinpoint** | **AAPC** | **ARIMAX** |
| Australia (145) | 1.3% | **0.9%** | 1.0% | **3.5%** | 9.2% | 9.8% |
| Canada (208) | 1.6% | **1.4%** | 1.9% | -4.6% | **1.5%** | 6.1% |
| Denmark (341) | **0.4%** | 0.7% | 0.7% | **9.9%** | 13.6% | 14.3% |
| Estonia (252) | 2.6% | **1.0%** | 3.2% | -23.4% | **-16.1%** | -29.1% |
| Finland (444) | 0.2% | 0.6% | **0.1%** | **0.0%** | 5.7% | -1.3% |
| France (186) | **3.3%** | 4.4% | 7.6% | **6.1%** | 9.8% | 7.2% |
| Germany (156) | 1.5% | 1.2% | **1.0%** | 20.0% | 18.4% | **14.3%** |
| Iceland (18) | 1.3% | **1.0%** | 1.7% | -9.5% | **-5.9%** | 15.2% |
| India (155) | **19.1%** | 31.2% | 22.6% | **33.1%** | 45.2% | 38.3% |
| Israel (434) | 0.5% | **0.3%** | 3.2% | -10.2% | **-4.1%** | 22.7% |
| Italy (286) | 1.1% | **1.1%** | 6.1% | 18.4% | **18.2%** | 43.4% |
| Japan (6,094) | **0.1%** | 0.2% | 0.6% | **0.9%** | 3.2% | 11.3% |
| Lithuania (636) | 1.0% | **0.3%** | 0.4% | -13.9% | -9.2% | **3.1%** |
| Norway (397) | **0.4%** | 0.5% | 0.9% | -7.4% | -5.2% | **0.7%** |
| Singapore (319) | 4.1% | **3.4%** | 4.2% | 21.9% | **20.0%** | 22.3% |
| Slovakia (599) | 1.1% | **0.4%** | 4.7% | -14.6% | **-8.0%** | -30.8% |
| Slovenia (310) | 0.9% | **0.5%** | 2.2% | 15.7% | **12.0%** | 27.4% |
| Spain (99) | **1.8%** | 2.1% | 2.6% | **21.9%** | 24.1% | 30.6% |
| Sweden (666) | 0.3% | **0.2%** | 1.0% | **-3.0%** | 10.3% | 19.5% |
| Switzerland (35) | 1.4% | 6.8% | **1.2%** | 10.2% | 30.2% | **-7.2%** |
| The Netherlands (109) | 4.8% | **3.8%** | 5.5% | 39.9% | **36.4%** | 43.6% |
| UK (848) | **0.7%** | 0.7% | 2.0% | **-0.7%** | 12.3% | 18.8% |
| USA (2976) | 0.7% | **0.7%** | 0.8% | -3.2% | -1.2% | **0.5%** |
| **Weighted Average** | **1.3%** | 1.7% | 2.1% | **10.1%** | 11.7% | 15.7% |

**Notes:**  **Abbreviations:** AAPC, average annual percentage change; ARIMAX, autoregressive integrated moving average with exogenous variables.

^a^ The projection errors were compared across the three methods, and the one with the least error was bolded.

^b^ As of the latest available year per registry.

**eTable 2. Comparison between Actual and 10-year Projected Incidence on Prostate Cancer**

| **Country**  **(Total incidence**^b^**)** | **Normalized Mean-squared-error**  **over the Period**^a^ | | | **Percentage Error**  **at the Tenth Year**^a^ | | |
| --- | --- | --- | --- | --- | --- | --- |
|  | **Joinpoint** | **AAPC** | **ARIMAX** | **Joinpoint** | **AAPC** | **ARIMAX** |
| Australia (1648) | 29.7% | 10.5% | **4.3%** | -75.7% | 17.6% | **-4.8%** |
| Canada (1581) | 9.5% | 3.1% | **2.6%** | 8.2% | 5.3% | **-1.1%** |
| Denmark (3091) | 5.8% | 1.2% | **1.0%** | -30.3% | **-10.4%** | -10.9% |
| Estonia (612) | 7.2% | **5.4%** | 9.3% | -33.0% | **-27.6%** | -39.6% |
| Finland (2909) | 0.4% | **0.3%** | 3.5% | 4.8% | **4.0%** | -22.7% |
| France (1572) | 15.2% | **5.4%** | 7.4% | -34.4% | **-17.7%** | -26.3% |
| Germany (647) | 15.5% | **8.4%** | 9.0% | -52.9% | **-39.6%** | -43.3% |
| Iceland (164) | 6.0% | **1.9%** | 6.8% | 39.7% | **19.5%** | -25.8% |
| India (266) | 38.2% | 36.7% | **13.9%** | 67.8% | 66.5% | **29.8%** |
| Israel (1927) | 4.6% | **1.8%** | 2.7% | **11.9%** | -19.5% | -25.9% |
| Italy (734) | 3.3% | **2.4%** | 3.5% | **5.3%** | 10.0% | -7.4% |
| Japan (2571) | **2.6%** | 5.1% | 12.5% | 16.4% | **10.0%** | -30.2% |
| Lithuania (2574) | **16.9%** | 31.4% | 36.4% | **-50.6%** | -66.3% | -71.1% |
| Norway (3068) | **0.1%** | 0.2% | 0.6% | 4.2% | **0.1%** | -9.6% |
| Singapore (384) | 4.8% | **1.7%** | 11.0% | 36.5% | **19.3%** | -35.3% |
| Slovakia (1173) | **0.3%** | 0.3% | 1.3% | -12.5% | **-2.3%** | -16.9% |
| Slovenia (745) | 6.4% | 1.2% | **1.0%** | 44.8% | 17.7% | **-4.4%** |
| Spain (277) | **1.2%** | 3.4% | 6.9% | **1.6%** | -8.8% | -25.9% |
| Sweden (6230) | 0.3% | 0.6% | **0.1%** | **-0.8%** | 6.3% | -5.5% |
| Switzerland (163) | 3.3% | 3.5% | **1.6%** | **2.0%** | 2.6% | -18.9% |
| The Netherlands (437) | **7.4%** | 8.2% | 9.6% | **-29.5%** | -31.1% | -35.4% |
| UK (3054) | 202.1% | **1.3%** | 1.6% | 216.6% | **3.6%** | -13.8% |
| US (27733) | 1229.0% | 51.4% | **18.2%** | 416.5% | 118.8% | **58.3%** |
| **Weighted Average**^c^ | 22.3% | **4.9%** | 5.3% | 73.2% | **23.4%** | 25.9% |

**Notes:** **Abbreviations:** AAPC, average annual percentage change; ARIMAX, autoregressive integrated moving average with exogenous variables.

^a^ The projection errors were compared across the three methods, and the one with the least error was bolded.

^b^ As of the latest available year per registry.

^c^ The incidences of prostate cancer from the United States were excluded from calculations of the weighted averages as all three methods produced extremely poor results and resulted in immense error rates.

**eTable 3. Comparison between Actual and Projected Cancer Incidence based on the Different Gender Groups**

| **Cancer**  **(Total incidence**^a^**)** | | **Normalized Mean-squared-error**  **over the Period** | | | | | | **Percentage Error** | | | | | | | | |  |
| --- | --- | --- | --- | --- | --- | --- | --- | --- | --- | --- | --- | --- | --- | --- | --- | --- | --- |
|  |  | **Joinpoint** | | **AAPC** | | **ARIMAX** | | | **Joinpoint** | | | | **AAPC** | | **ARIMAX** | | |
|  |  | **F** | **M** | **F** | **M** | **F** | **M** | | **F** | | | **M** | **F** | **M** | **F** | **M** | |
| **In 3-year Projection** | | | | | | | | | | | | | | | | |  |
| Bladder (31 648) | | 3.8% | 4.3% | 2.9% | 3.2% | 2.3% | 2.8% | | 6.1% | 6.8% | | | 5.0% | 5.5% | 1.1% | 1.2% | |
| CRC (85 600) | | 0.9% | 0.8% | 0.6% | 0.7% | 1.2% | 1.2% | | 3.9% | 3.6% | | | 3.1% | 3.5% | 1.7% | 1.8% | |
| Esophagus (9597) | | 4.5% | 4.3% | 2.6% | 2.4% | 2.7% | 2.6% | | 4.0% | 3.8% | | | 3.5% | 3.2% | 1.9% | 1.9% | |
| Lung (83767) | | 0.9% | 0.9% | 0.8% | 0.7% | 0.6% | 0.7% | | 4.7% | 4.7% | | | 5.1% | 4.6% | 1.2% | 1.3% | |
| Pancreas (19 823) | | 2.1% | 1.9% | 1.3% | 1.1% | 1.3% | 1.2% | | 2.3% | 2.0% | | | 0.4% | 0.3% | -2.6% | -2.6% | |
| Prostate (68 536) | | 4.1% | 4.3% | 2.5% | 2.3% | 2.3% | 2.2% | | -1.9% | -2.0% | | | -4.0% | -3.8% | -9.0% | -8.5% | |
| Stomach (22 613) | | 1.3% | 1.2% | 1.4% | 1.6% | 1.4% | 1.2% | | 0.8% | 0.7% | | | 4.5% | 4.7% | 4.5% | 4.1% | |
| **Weighted Average** | | 2.3% | 2.1% | 1.5% | 1.5% | 1.6% | 1.6% | | 3.6% | 3.6% | | | 3.9% | 3.9% | 3.3% | 3.3% | |
|  | |  | |  | |  | | |  | | | |  | |  | | |
| **In 5-year Projection** | |  | |  | |  | | |  | | | |  | |  | | |
| Bladder (31 648) | | 6.3% | 6.7% | 4.7% | 4.6% | 3.6% | 3.9% | | 15.2% | | 16.2% | | 14.1% | 13.8% | 7.7% | 8.1% | |
| CRC (85 600) | | 1.3% | 1.1% | 0.9% | 1.0% | 1.5% | 1.4% | | 4.1% | | 3.5% | | 3.8% | 4.2% | 0.8% | 0.8% | |
| Esophagus (9597) | | 5.7% | 6.2% | 2.6% | 2.5% | 3.2% | 2.7% | | 2.1% | | 2.8% | | 1.6% | 1.4% | 0.3% | 0.2% | |
| Lung (83 767) | | 1.6% | 1.5% | 1.2% | 1.0% | 0.9% | 0.8% | | 7.4% | | 7.7% | | 10.1% | 8.5% | 3.4% | 3.0% | |
| Pancreas (19 823) | | 2.7% | 2.5% | 1.7% | 1.2% | 1.4% | 1.7% | | 1.1% | | 0.9% | | -0.4% | -0.3% | -4.7% | -5.0% | |
| Prostate (68 536) | | 8.0% | 8.6% | 3.2% | 2.8% | 3.0% | 2.8% | | 1.1% | | 1.2% | | -0.5% | -0.5% | 8.6% | 8.0% | |
| Stomach (22 613) | | 1.4% | 1.4% | 1.5% | 1.6% | 1.9% | 1.8% | | 1.0% | | 1.0% | | 4.6% | 4.9% | 6.8% | 6.5% | |
| **Weighted Average** | | 3.5% | 3.6% | 1.9% | 1.8% | 1.9% | 1.9% | | 6.5% | | 6.7% | | 6.8% | 6.8% | 4.8% | 5.0% | |
|  | |  | |  | |  | | |  | | | |  | |  | | |
| **In 10-year Projection** | |  | |  | |  | | |  | | | |  | |  | | |
| Bladder (21 411) | | 10.4% | 10.7% | 5.2% | 5.6% | 2.8% | 3.0% | | 22.7% | | 23.2% | | 19.6% | 20.2% | 3.0% | 3.1% | |
| CRC (53 414) | | 2.3% | 2.2% | 1.3% | 1.4% | 1.9% | 1.9% | | 8.4% | | 8.3% | | 9.9% | 10.3% | 1.7% | 1.6% | |
| Esophagus (5541) | | 14.7% | 14.9% | 3.4% | 3.2% | 3.7% | 3.4% | | 6.2% | | 6.3% | | 3.1% | 2.7% | 0.3% | 0.2% | |
| Lung (55 601) | | 3.5% | 3.8% | 4.7% | 4.5% | 1.6% | 1.6% | | 22.3% | | 23.4% | | 31.5% | 30.2% | 8.2% | 8.1% | |
| Pancreas (13 344) | | 4.4% | 4.7% | 2.9% | 2.8% | 2.3% | 2.0% | | 12.9% | | 13.8% | | 12.3% | 12.0% | 1.1% | 0.7% | |
| Prostate^b^ (35 827) | | 25.9% | 26.7% | 5.5% | 5.6% | 6.5% | 6.4% | | 21.8% | | 23.1% | | 1.7% | 1.7% | -18.9% | -18.4% | |
| Stomach (15 713) | | 1.4% | 1.5% | 1.7% | 1.7% | 2.2% | 2.1% | | 0.1% | | 0.1% | | 4.5% | 4.5% | 8.0% | 7.8% | |
| **Weighted Average** | | 8.1% | 8.3% | 3.6% | 3.6% | 2.8% | 2.8% | | 15.8% | | 16.3% | | 15.0% | 14.8% | 7.1% | 6.9% | |

**Notes:** **Abbreviations:** AAPC, average annual percentage change; ARIMAX, autoregressive integrated moving average with exogenous variables.

^a^ As of the latest available year per registry. 66 registries were included for 3-year and 5-year projections while 41 registries were included for 10-year projections, resulting in different fewer total incidences for 10-year projection.

^b^ The incidences of prostate cancer from the United States were excluded in 10-year projection as all three methods produced poor results and resulted in exceptionally large error rates.
